# Factors affecting patient satisfaction related to cost and treatment effectiveness in rheumatoid arthritis: results from the multicenter observational cohort study, FRANK Registry

**DOI:** 10.1186/s13075-022-02746-5

**Published:** 2022-02-22

**Authors:** Toshifumi Fujiwara, Masakazu Kondo, Hisakata Yamada, Akihisa Haraguchi, Kenjiro Fujimura, Koji Sakuraba, Satoshi Kamura, Jun-ichi Fukushi, Hisaaki Miyahara, Yasushi Inoue, Tomomi Tsuru, Toshihide Shuto, Seiji Yoshizawa, Eiichi Suematsu, Tomoya Miyamura, Masahiro Ayano, Hiroki Mitoma, Yojiro Arinobu, Hiroaki Niiro, Masanobu Ohishi, Akie Hirata, Shoji Tokunaga, Atsushi Takada, Daisuke Hara, Hidetoshi Tsushima, Yukio Akasaki, Satoshi Ikemura, Takuya Sueishi, Masakazu Toya, Takahide Sakuragi, Tomoko Tsutsui, Kazuhiro Kai, Shinkichi Arisumi, Yasuharu Nakashima

**Affiliations:** 1 Department of Orthopaedic Surgery, Kyushu University Graduate School of Medical Sciences, 3-1-1, Maidashi, Higashi-ku, Fukuoka, 812-8582 Japan; 2 Kondo Clinic of Rheumatology and Orthopaedic Surgery, Fukuoka, Japan; 3 Department of Orthopedics Surgery and Rheumatology, National Hospital Organization Kyushu Medical Center, Fukuoka, Japan; 4 Department of Rheumatology, Fukuoka Red Cross Hospital, Fukuoka, Japan; 5 PS Clinic, Fukuoka, Japan; 6 Department of Orthopedics Surgery, Chiyoda Hospital, Miyazaki, Japan; 7 Department of Rheumatology, Hamanomachi Hospital, Fukuoka, Japan; 8 Department of Internal Medicine and Rheumatology, National Hospital Organization Kyushu Medical Center, Fukuoka, Japan; 9 Department of Medicine and Biosystemic Science, Kyushu University Graduate School of Medical Sciences, Fukuoka, Japan; 10 Department of Medical Education, Kyushu University Graduate School of Medical Sciences, Fukuoka, Japan; 11 Department of Orthopaedic Surgery, Chihaya Hospital, Fukuoka, Japan; 12 Medical Information Center, Kyushu University Hospital, Fukuoka, Japan

**Keywords:** Rheumatoid arthritis, Patient satisfaction, Quality of life, Observational study, Remission, Low disease activity

## Abstract

**Background:**

To further improve rheumatoid arthritis (RA) treatment, it is necessary to understand each RA patient’s satisfaction and to identify the factors affecting their satisfaction. Despite the rise in medical costs for RA, little is known about the factors that influence patient satisfaction with the cost of treatment in RA patients.

**Methods:**

This is a multicenter observational study of Japanese RA patients from the FRANK Registry with data analyzed from March 2017 to August 2020. We collected data on demographic characteristics, clinical data, quality of life which was evaluated using the EuroQol 5-dimensional questionnaire (EQ5D), and patient satisfaction. The four categories of patient satisfaction were evaluated individually (i.e., cost, treatment efficacy, activities of daily living [ADL], and global treatment satisfaction). We analyzed the factors that affected each patient’s satisfaction, such as age, sex, EQ5D, disease duration, disease activity, and treatment.

**Results:**

This study included 2235 RA outpatients (406 males, 1829 females). In RA patients, “very satisfied” and “satisfied” were given for nearly half of each satisfaction aspect (cost 49%; efficacy 72%; ADL 58%; global treatment 66%) at the time of the initial registration. To investigate the factors influencing each satisfaction, multivariate analysis has revealed that the use of b/tsDMARDs increased satisfaction of treatment effect (odds ratio [OR] 0.66) and ADL (OR 0.78) but decreased cost satisfaction (OR 2.21). Age (50–64 years; OR 0.91; 65–74 years, 0.55: ≥ 75 years, 0.35), female (OR 0.81), and history of musculoskeletal surgery (OR 0.60) all increased cost satisfaction. Patients with lower disease activity and higher EQ5D scores had higher levels of satisfaction in all areas.

**Conclusions:**

In this study, patient satisfaction in terms of cost, treatment effect, ADL, and overall treatment was generally higher, but some patients were dissatisfied. The cost of satisfaction increased with age and a history of musculoskeletal surgery, while it decreased with a lower EQ5D score and the use of b/tsDMARDs.

**Supplementary Information:**

The online version contains supplementary material available at 10.1186/s13075-022-02746-5.

## Introduction

Rheumatoid arthritis (RA) is a progressive inflammatory autoimmune disease that primarily affects the joints and potentially impairs the patient’s quality of life (QOL). In the last decade, a paradigm shift has been seen in the treatment of RA, the so-called treat-to-target (T2T) strategy, which involves more aggressive, tightly controlled therapy early in the disease course guided by a structured assessment of disease activity, with the ultimate goal of reaching remission [1]. The advances in understanding the pathogenesis of RA have given rise to new therapeutic target agents. Nowadays, there are several options for the combination of disease-modifying anti-rheumatic drugs (DMARDs), including conventional synthetic DMARDs (csDMARDs), biologic DMARDs (bDMARDs), and targeted synthetic DMARDs (tsDMARDs) [2]. These improvements in treatment could dramatically alter the patients’ QOL and ability to carry out activities of daily living (ADL).

The recent availability of effective therapies has been linked with beneficial outcomes and improved QOL in RA patients, but long-lasting pharmacological treatment may become a chronic burden, especially regarding cost and safety concerns. In the T2T approach for RA treatment, it is necessary that patients comprehend the T2T recommendations and properly communicate with their physicians. However, several reports have presented discordances between patients and physicians regarding their opinions on treatment outcomes, satisfaction, and ADL [3–7]. Increased patient satisfaction has influenced RA treatment adherence and continuation [8, 9], and this has been enhanced by successful treatments decided on by both the patient and physician [10, 11]. Thus, understanding patient satisfaction could lead to the overall improvement of RA treatment. Treatment satisfaction of patients with RA has reportedly been improved by the use of bDMARDs, low disease activity, and better communication with their physicians, whereas satisfaction was reduced by unfavorable treatment costs [12, 13]. High treatment costs have been found to reduce patient satisfaction, but it is critical to further characterize patient satisfaction in terms of treatment effectiveness and cost separately following the T2T strategy and to eventually improve treatment adherence [14–17]. So far, no reports have examined RA patient satisfaction in terms of cost and treatment effectiveness in a large cohort study.

Using the initial registration data from a regional observational cohort registry, we assessed RA patient satisfaction in terms of cost, treatment effectiveness, current ADL, and overall treatment in the current study.

This study aimed at identifying factors influencing satisfaction in RA patients, as well as to investigate factors associated with each dimension of patient satisfaction to describe various aspects of RA treatment.

## Materials and methods

### Data collection

In 2018, we launched a multicenter prospective observational cohort study of RA patients at Kyushu University, using data from the Fukuoka Rheumatoid Arthritis Network (FRANK) Registry. The Kyushu University ethics committee approved the FRANK Registry (approval number: 29-277). The FRANK Registry includes data from a total of nine associated institutions (Kyushu University, Kondo Clinic of Rheumatology and Orthopedic Surgery, National Hospital Organization, Kyushu Medical Center, Fukuoka Red Cross Hospital, PS Clinic, Chiyoda Hospital, Hamanomachi Hospital, and Chihaya Hospital), including clinics and hospitals located in the suburbs of Fukuoka Prefecture in the south of Japan since March 2018. Clinical data were submitted to the data center in Kyushu University Hospital through the Clinical Research Internet Network (CRIN-Q) [18]. After obtaining informed consent, we enrolled each RA patient during his/her clinic or hospital visits. Our study included Japanese patients over the age of 18 who were diagnosed with RA using the 1987 American College of Rheumatology (ACR) classification criteria. These RA patients were treated by rheumatologists who followed the guidelines of the Japan College of Rheumatology. Each year, the rheumatologists gathered the following demographic and clinical data from each patient: age, sex, body mass index, job, disease duration, Steinbrocker stage, physician visual analog scales (VAS), tender 28-joint count (TJC), swollen 28-joint count (SJC), C-reactive protein (CRP) level, erythrocyte sedimentation ratio (ESR), rheumatoid factor positivity, anti-cyclic citrullinated peptide antibody positivity, treatment, and comorbidities (e.g., cardiovascular disorder, a pulmonary disorder, osteoporosis, and malignancy). Steinbrocker stage was determined by radiographic abnormalities of the hand in patients with RA as follows: stage I, no destructive changes; stage II, slight cartilage and/or subchondral bone destruction and osteoporosis; stage III, cartilage and/or bone destruction and osteoporosis; and stage IV, osseous ankylosis [19, 20]. Regarding the present and past treatments, we analyzed the patients’ medication use and history of musculoskeletal surgery, such as the use and dose of csDMARDs (e.g., methotrexate), the use and dose of prednisolone, the use of b/tsDMARDs, the number of previously administered b/tsDMARDs, the reasons for discontinuation or non-use of b/tsDMARDs, and the history of musculoskeletal surgery associated with RA, such as arthroplasty [21, 22]. The enrolled patients were asked to complete the self-administered questionnaires each year. The questionnaires consisted of pain VAS and general VAS, Modified Health Assessment Questionnaire (mHAQ) [23], EuroQol 5-Dimensional Questionnaire (EQ5D) [24], and patient satisfaction questionnaires. The utility index scores of the EQ5D were calculated using the Japanese version of the EQ5D [25]. We separated the subjective variables evaluated by RA patients (i.e., pain VAS, general VAS, and EQ5D score) and the objective variables assessed by a physician (i.e., TJC, SJC, log-transformed CRP, physician VAS, history of musculoskeletal surgery, a dose of prednisolone, use of methotrexate, and use of b/tsDMARDs) in this study to investigate the factors influencing each satisfaction showing the subjective result.

### Patient satisfaction questionnaire contents

RA patient satisfaction with treatment effectiveness and with tolerability was established to measure in a 4-point scale (very satisfied, rather satisfied, rather unsatisfied, and very unsatisfied) [10, 11], and we segmented these scales of satisfaction into 6-point Likert scales with the response [26–29]. Each self-administered satisfaction questionnaire was assessed using a 6-point scale as follows: very satisfied (1 point), satisfied (2 points), somewhat satisfied (3 points), somewhat unsatisfied (4 points), unsatisfied (5 points), and very unsatisfied (6 points). Furthermore, patient satisfaction was individually assessed in terms of the following four categories: (1) cost of treatment, (2) effect of treatment, (3) ADL, and (4) global treatment.

### Patients included in the analysis

**Fig. 1:**
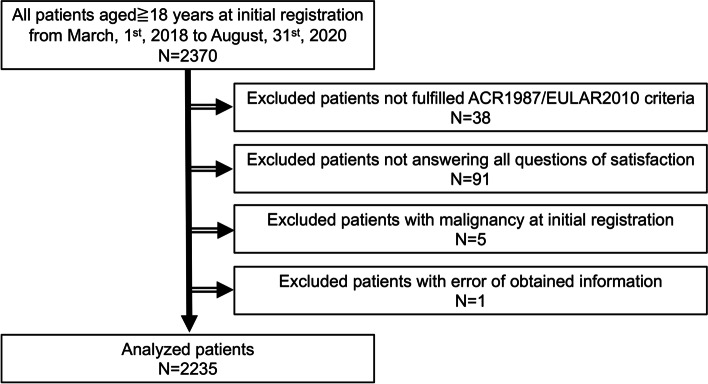
Flowchart of the selection of rheumatoid arthritis patients from the initial registration data of the FRANK Registry

Between March 2018 and August 2020, a total of 2370 patients were registered on the FRANK Registry. This was a cross-sectional analysis of information collected and registered by August 2020. At the time of their initial registration, all patients were 18 or older. The following patients were excluded: (1) participants who did not fulfill the 2010 ACR/European League Against Rheumatism and the 1987 ACR criteria for RA (*N* = 38), (2) those who lacked data on the four-dimension satisfaction questionnaires (*N* = 91), (3) those with malignancy upon initial registration (*N* = 5), and (4) patients with errors in their obtained information (*N* = 1). After exclusion, the data of 2235 patients were analyzed in this study (Fig. 1). To examine the test-retest reliability of each satisfaction survey, 68 out of 2235 patients were randomly selected and were required to score each aspect of satisfaction during two consecutive visits within 3 months.

### Statistical analysis

Descriptive statistics were used to summarize the characteristics of the patients included in the study. Because this cohort included the majority of patients with low disease activity and remission (Table 1), the normal distribution was skewed when statistically analyzing the dichotomizing measurement data. An ordered logistic regression model was used to analyze the association between satisfaction score and the following variables: age (i.e., < 50, 50–64, 65–74, and ≥ 75 years), sex, RA disease duration (dichotomized at median value), subjective variables (i.e., pain VAS [cm] dichotomized at median value, general VAS [cm] dichotomized at a median value, and EQ5D score categorized in quartiles), and objective variables (i.e., TJC [0 or ≥ 1], SJC [0 or ≥ 1], log-transformed CRP [mg/dL] as a continuous variable, physician VAS [cm] dichotomized at a median value, history of musculoskeletal surgery, a dose of prednisolone [over 5 mg or not], and use of b/tsDMARDs). First, we confirmed the association between these patient satisfaction and subjective variables. Afterward, we analyzed the association between these aspects of satisfaction and objective variables. Two separate models (subjective and objective) were used in the multivariate ordered logistic regression analysis [30] to assess the factors associated with each aspect of satisfaction as explanatory variables; all models included age, sex, and disease duration as common covariates. The odds ratio (OR) was set to increase according to dissatisfaction. To analyze the interactions of each variable, we examined the statistical significance of the interactions between two of the dichotomous explanatory variables, adding product terms of all pairs of the two covariates into the multivariate model using instrumental variable analysis (Table 6). To evaluate the statistical significance of the interaction between the EQ5D score (four indicator variables) and one of the dichotomous covariates, a likelihood ratio test was used, comparing a multivariate model that included the four product terms to a model without them. Test-retest reliability was assessed using kappa statistics. The association of each satisfaction was measured using Spearman correlation coefficients. SAS version 9.4 was used to conduct all statistical analyses (SAS Institute, Inc., Cary, NC, USA).

**Table 1:** Baseline characteristics of the included patients

Variables	*N* = 2235
Age (years) (median, range)	65, 19–98
<50 (%)	381 (17%)
50–64 (%)	699 (31%)
65–74 (%)	767 (34%)
≥ 75 (%)	388 (17%)
Sex (male, female) (%)	406 (18%), 1829 (82%)
Body mass index (median, range)	22, 13–42
Job (yes, no) (%)	957 (45%), 1179 (55%)
Disease duration (years) (median, range)	11, 0–70
Steinbrocker stage 1, 2, 3, 4 (%)	612 (27%), 709 (32%), 305 (14%), 560 (25%)
Comorbidities	
Cardiovascular disorder	106 (5%)
Pulmonary disorder	193 (9%)
Osteoporosis	285 (23%)
TJC (median, range)	0, 0–28
SJC (median, range)	0, 0–13
RF (positive, negative)	1574 (73.7%), 563 (26.4%)
ACPA (positive, negative)	1541 (78.9%), 411 (21.1%)
CRP (mg/dL) (median, range)	0.11, 0–21.9
ESR 1 h (mm) (median, range)	14, 0–144
Treatment	
Medication	
Prednisolone use (yes, no) (%)	896 (40%), 1339 (60%)
Dose (mg) (median, range)	3, 0–18
Methotrexate use (yes, no) (%)	1614 (72%), 621 (28%)
Dose (mg) (median, range)	8, 2–20
b/tsDMARDs use (yes, no) (%)	697 (31%), 1538 (69%)
History of musculoskeletal surgery (yes, no) (%)	463 (21%), 1769 (79%)
Prosthesis	246 (11%)
Postoperative < 5 years, ≥ 5 years	77 (31%), 169 (69%)
Spine surgery	48 (2%)
Postoperative < 5 years, ≥ 5 years	15 (31%), 33 (69%)
Others*	186 (8%)
Postoperative < 5 years, ≥ 5 years	61 (33%), 110 (59%)
Pain VAS (cm) (median, range)	1.2, 0–10
General VAS (cm) (median, range)	0.9, 0–10
Physician VAS (cm) (median, range)	0.5, 0–10
DAS28-ESR	2.3, 0–7.1
Remission (≤ 2.6)	1298 (61%)
Low disease activity (> 2.6, ≤ 3.2)	788 (18%)
Moderate disease activity (> 3.2, ≤ 5.1)	188 (20%)
High disease activity (> 5.1)	16 (1%)
SDAI	2.9, 0–43.3
Remission (≤ 3.3)	1175 (54%)
Low disease activity (> 3.3, ≤ 11)	788 (36%)
Moderate disease activity (> 11, ≤ 26)	188 (9%)
High disease activity (> 26)	16 (1%)
CDAI	2.7, 0–39.5
Remission (≤ 2.8)	1113 (51%)
Low disease activity (> 2.8, ≤ 10)	853 (39%)
Moderate disease activity (> 10, ≤ 22)	184 (8%)
High disease activity (> 22)	18 (1%)
mHAQ (median, range)	0.1, 0–3.0
EQ5D score (median, range)	0.8, 0.03–1.0

Other history of musculoskeletal surgery included arthrodesis, arthroplasty, synovectomy, surgery for tendon, or surgery associated with fracture

*TJC* tender 28-joint count, *SJC* swollen 28-joint count, *RF* rheumatoid factor, *ACPA*, anti-citrullinated protein/peptide antibody, *CRP* C-reactive protein, *ESR* erythrocyte sedimentation ratio, *b/tsDMARDs* biological/targeted synthetic disease-modifying antirheumatic drugs, *VAS* visual analog scales, *DAS28-ESR* disease activity score 28-ESR, *SDAI* Simple Disease Activity Index, *CDAI* Clinical Disease Activity Index, *mHAQ* Modified Health Assessment Questionnaire, *EQ5D* EuroQol 5 Dimensions

## Results

### Patient characteristics

**Fig. 2:**
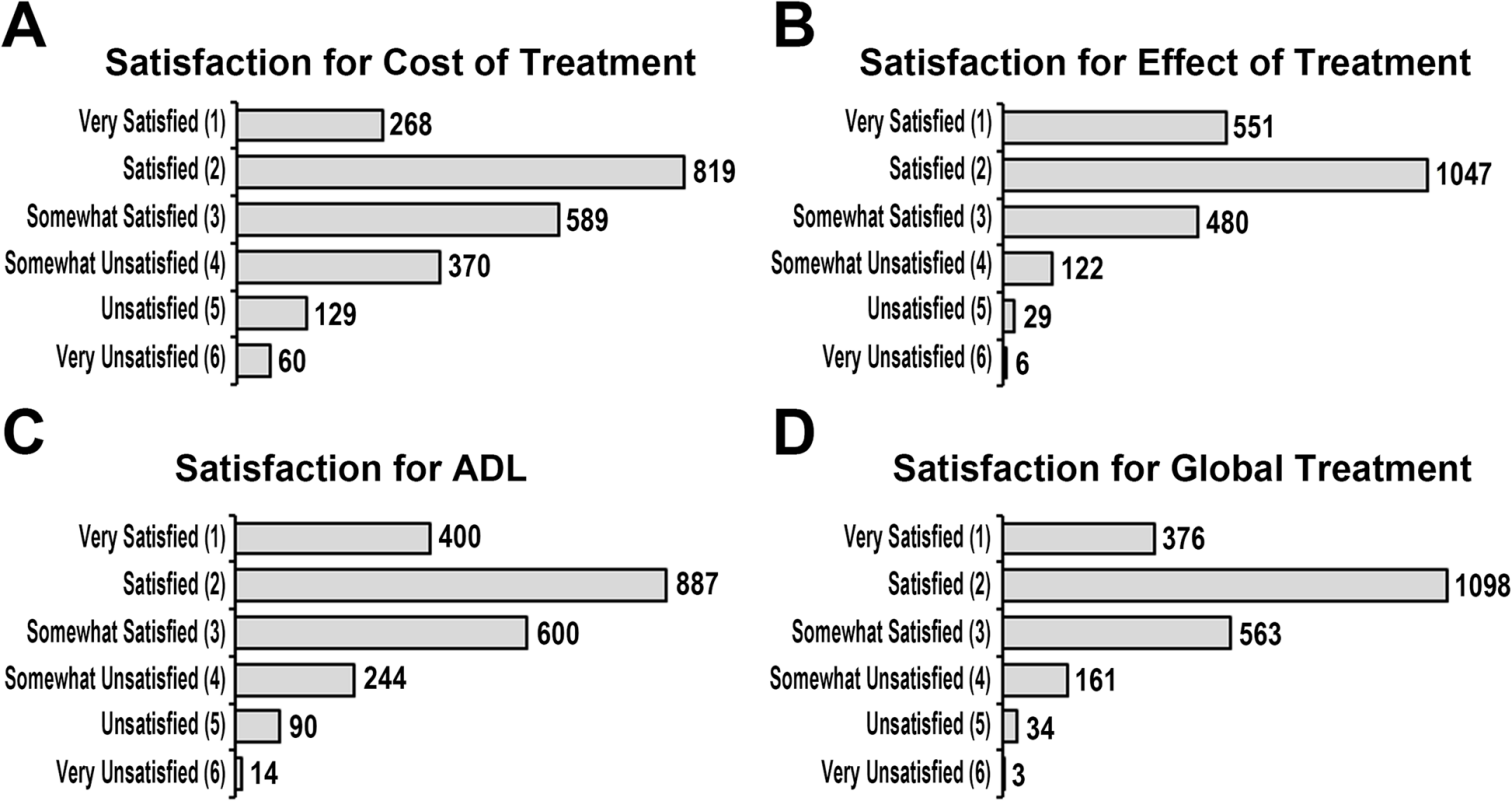
Distribution of satisfaction ratings from the initial registration data of the FRANK Registry. Each self-administered satisfaction rating was evaluated via a 6-step score (very satisfied, 1 point; satisfied, 2 points; somewhat satisfied, 3 points; somewhat unsatisfied, 4 points; unsatisfied, 5 points; very unsatisfied, 6 points). **A** Satisfaction for the cost of treatment. **B** Satisfaction for the effect of treatment. **C** Satisfaction for ADL. **D** Satisfaction for global treatment

**Fig. 3:**
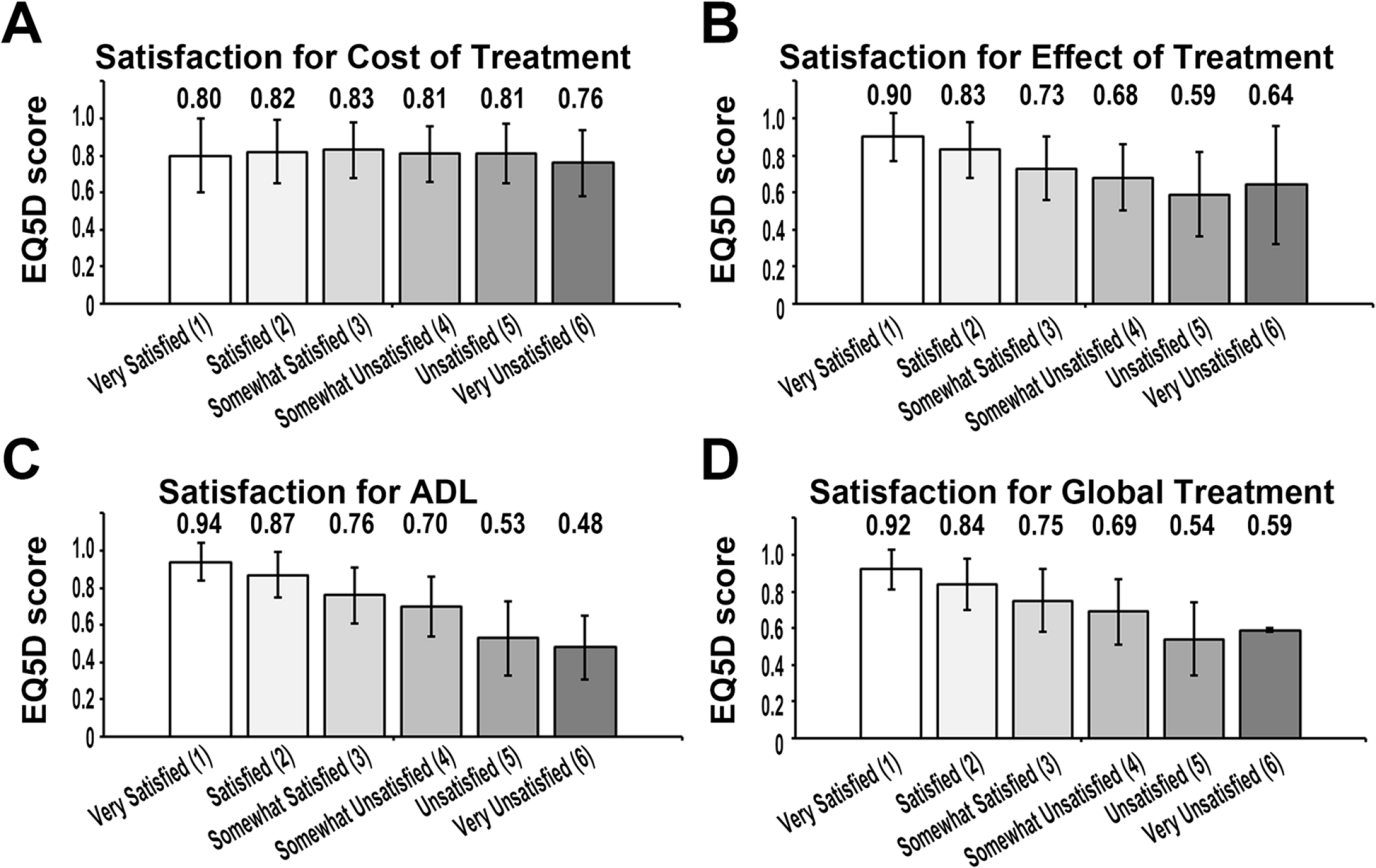
Distribution of EQ5D score in each satisfaction rating from the initial registration data of the FRANK Registry. **A** EQ5D score in satisfaction for the cost of treatment. **B** EQ5D score in satisfaction for the effect of treatment. **C** EQ5D score in satisfaction for ADL. **D** EQ5D score in satisfaction for the global treatment

A total of 2235 Japanese patients with RA from the FRANK Registry were evaluated in this study (Fig. 1). Table 1 shows the baseline characteristics and clinical information. The median age and disease duration at the time of initial registration were 65 (range 19–98) years and 11 (range 0–70) years, respectively. Each median TJC, SJC, serum CRP, and ESR were 0 (range 0–28), 0 (range 0–13), 0.11 (range 0–21.9) mg/dL, and 14 (range 0–10) mm/h, respectively, indicating that almost all patients had lower disease activity in this cohort. Prednisolone was administered to 896 patients (40 percent), methotrexate was administered to 1614 patients (72%), and b/tsDMARDs were administered to 697 patients (31%). The patients with a history of musculoskeletal surgery were 463 patients (21%), and 246 patients of whom (11%) had undergone joint prosthesis. The patients undergone spine surgery or others including arthrodesis, arthroplasty, synovectomy, surgery for tendon, or surgery associated with fracture were 48 patients (2%) or 186 patients (8%), respectively. The patients having surgery for joint prosthesis less than 5 years were 77 patients (31%), and 169 patients (69%) had been over 5 years. The median disease activity score 28-ESR (DAS28-ESR), simple disease activity index, and clinical disease activity index were 2.3 (range 0–7.1), 2.9 (range 0–43.3), and 2.7 (range 0–39.5), respectively. The patients with low disease activity and remission have consisted of over 80% of this cohort. The median mHAQ score was 1.1 (range 1–4), while the median EQ5D score was 0.8 (range 0.03–1.0). Figure 2 depicts the distributions for each aspect of satisfaction. For nearly half of each satisfaction aspect, scores of “very satisfied” (1 point) and “satisfied” (2 points) were assigned (cost of treatment, 49%; effect of treatment, 72%; ADL, 58%; global treatment, 66%). Figure 3 depicts the mean ± standard deviation of EQ5D score in scores (i.e., “very satisfied,” “satisfied,” “somewhat satisfied,” “somewhat unsatisfied,” “unsatisfied,” and “very unsatisfied”) of each satisfaction (i.e., cost of treatment, the effect of treatment, ADL, and global treatment). Internal reliability of patient-estimated satisfaction was analyzed using kappa statistics: 0.75 for cost, 0.53 for treatment effect, 0.60 for ADL, and 0.58 for global treatment. Because these surveys for each satisfaction were obtained independently, we have not evaluated the combined analysis of each satisfaction. Additional file 1: Table S1 shows the correlation coefficients for each satisfaction. Satisfaction for the cost of treatment had a weaker relationship with other satisfactions (effect of treatment: *r* = 0.32; ADL: *r* = 0.25; global treatment: *r* = 0.43); on the other hand, satisfaction for the effect of treatment, ADL, and global treatment presented a strong relationship with each other (effect of treatment and ADL: *r* = 0.69; effect of treatment and global treatment: *r* = 0.76; ADL and global treatment: *r* = 0.74).

### Factors associated with satisfaction with cost of treatment

Table 2 shows the univariate and multivariate analyses of the subjective and objective factors associated with satisfaction of cost of treatment. Compared with the patients under the age of 50 years old as a reference, satisfaction with the cost of treatment improved with aging in both subjective (50–64 years: OR 0.78 [*p* = 0.037]; 65–74 years: OR 0.43 [*p* < 0.001]; ≥ 75 years: OR 0.25 [*p* < 0.001]) and objective (65–74 years: OR 0.55 [*p* < 0.001]; ≥ 75 years: OR 0.35 [*p* < 0.001]) factors. Having lower EQ5D score significantly increased dissatisfaction with the cost of treatment (0.9–1.0, referent; 0.85–0.89: OR 1.52 [*p* = 0.004]; 0.75–0.84: OR 1.54 [*p* < 0.001]; 0–0.74: OR 1.27 [*p* = 0.044]). In the multivariate analysis of objective factors, the presence of SJC (OR 1.36 [*p* = 0.002]), higher physician VAS (> 0.5; OR 1.33 [*p* = 0.003]), and the use of b/tsDMARDs (OR 2.21 [*p* < 0.001]) significantly increase dissatisfaction. Interestingly, higher CRP level (OR 0.92 [*p* = 0.006]) and having a higher history of musculoskeletal surgery (OR 0.60 [*p* < 0.001]) significantly increased satisfaction. Each level of serum CRP and medication (i.e., prednisolone use and dose, methotrexate use and dose, and b/tsDMARDs use) in each score of satisfaction of cost of treatment is presented in Additional file 1: Table S2. The kinds of musculoskeletal surgery (i.e., prosthesis, spine surgery, and others) and postoperative duration (< 5 years, ≥ 5 years) in each score of satisfaction of cost of treatment were shown in Additional file 1: Table S3. The comorbidities (i.e., cardiovascular disorder, a pulmonary disorder, and osteoporosis) in each score of satisfaction of cost of treatment were shown in Additional file 1: Table S5. In the subjective model, significant interactions were observed in disease duration, pain VAS, and EQ5D. Additionally, significant interactions in the objective model were seen for age, sex, disease duration, physician VAS, CRP, history of musculoskeletal surgery, and use of b/tsDMARDs (Table 6).

**Table 2:** Factors associated with satisfaction with treatment cost

		Univariate			Multivariate					
					Subjective model			Objective model		
		OR	95% CI	*p*-value	OR	95% CI	*p*-value	OR	95% CI	*p*-value
Common variables										
Age (years)	< 50	Ref	–	–	Ref	–	–	Ref	–	–
	50–64	0.77	0.62–0.97	0.023	0.78	0.62–0.99	0.037	0.91	0.71–1.15	0.410
	65–74	0.45	0.36–0.57	< 0.001	0.43	0.34–0.54	< 0.001	0.55	0.44–0.70	< 0.001
	≥ 75	0.27	0.21–0.35	< 0.001	0.25	0.19–0.33	< 0.001	0.35	0.27–0.47	< 0.001
Sex	Female	0.97	0.80–1.18	0.791	0.86	0.70–1.05	0.138	0.81	0.65–0.99	0.041
Disease duration (years)	≥ 11	0.89	0.77–1.04	0.138	1.02	0.87–1.19	0.841	1.06	0.88–1.28	0.549
Subjective variables										
Pain VAS (cm)	> 1.2	1.34	1.15–1.554	< 0.001	1.19	0.98–1.45	0.088	–	–	–
General VAS (cm)	> 0.9	1.16	1.00–1.35	0.047	1.01	0.84–1.23	0.900	–	–	–
EQ5D score	0.9–1.0	Ref	–	–	Ref	–	–	–	–	–
	0.85–0.89	1.51	1.14–2.00	0.004	1.52	1.14–2.03	0.004	–	–	–
	0.75–0.84	1.49	1.22–1.81	< 0.001	1.54	1.24–1.90	< 0.001	–	–	–
	0–0.74	1.17	0.97–1.42	0.109	1.27	1.01–1.59	0.044	–	–	–
Objective variables										
TJC	≥ 1	1.31	1.12–1.53	< 0.001	–	–	–	1.04	0.86–1.27	0.665
SJC	≥ 1	1.42	1.28–1.66	< 0.001	–	–	–	1.36	1.12–1.66	0.002
Log-transformed CRP (mg/dL)	Continuous	0.88	0.84–0.93	< 0.001	–	–	–	0.92	0.87–0.98	0.006
Physician VAS (cm)	> 0.5	1.26	1.08–1.46	0.003	–	–	–	1.33	1.10–1.61	0.003
Steinbrocker stage	3 and 4	0.87	0.74–1.01	0.069	–	–	–	0.84	0.69–1.03	0.100
Musculoskeletal surgery	Yes	0.57	0.47–0.69	< 0.001	–	–	–	0.60	0.48–0.74	< 0.001
Prednisolone dose (mg)	> 5	1.14	0.81–1.62	0.454	–	–	–	1.16	0.81–1.68	0.413
Methotrexate use	Yes	0.95	0.80–1.12	0.515	–	–	–	0.90	0.76–1.08	0.256
b/tsDMARDs use	Yes	2.47	2.10–2.91	< 0.001	–	–	–	2.21	1.85–2.65	< 0.001

*OR* odds ratio, *95% CI* 95% confidence interval

### Factors associated with satisfaction with the effect of treatment

As shown in Table 3, compared with patients under the age of 50 years old, satisfaction with the effect of treatment decreased with aging (50–64 years) (OR 1.41 [*p* = 0.006] and OR 1.30 [*p* = 0.037]), high pain VAS (OR 2.63 [*p* < 0.001]) and general VAS (OR 1.28 [p = 0.020]), lower EQ5D score (0.85–0.89: OR 1.90 [*p* < 0.001]; 0.75–0.84: OR 2.89 [*p* < 0.001]; 0–0.74: OR 4.55 [*p* < 0.001]), presence of TJC (OR 1.75 [*p* < 0.001]) and SJC (OR 1.41 [*p* < 0.001]), higher CRP level (OR 1.10 [*p* = 0.002]), elevated physician VAS (OR 2.21 [*p* < 0.001]), and Steinbrocker stages 3 and 4 (OR 1.32 [*p* = 0.010]). The use of methotrexate (OR 0.80 [*p* = 0.0145]) and b/tsDMARDs (OR 0.66 [*p* < 0.001]) improved satisfaction with the effect of treatment. The comorbidities in each score of satisfaction of effect of treatment were shown in Additional file 1: Table S5. Interactions in the subjective model were significantly seen for disease duration, pain VAS, and EQ5D, while interactions in the objective model were observed for TJC and a history of musculoskeletal surgery (Table 6).

**Table 3:** Factors associated with satisfaction with treatment effect

		Univariate			Multivariate					
					Subjective model			Objective model		
		OR	95% CI	*p*-value	OR	95% CI	*p*-value	OR	95% CI	*p*-value
Common variables										
Age (years)	< 50	Ref	–	–	Ref	–	–	–	–	–
	50–64	1.55	1.23–1.96	< 0.001	1.41	1.10–1.79	0.006	1.30	1.02–1.66	0.037
	65–74	1.23	0.74–1.54	0.082	1.07	0.84–1.36	0.606	0.95	0.74–1.22	0.683
	≥ 75	1.26	0.97–1.64	0.083	0.89	0.67–1.18	0.409	0.96	0.7–1.18	0.299
Sex	Female	1.08	0.88–1.32	0.454	0.89	0.72–1.10	0.288	1.07	0.86–1.33	0.572
Disease duration (years)	≥ 11	1.17	1.00–1.32	0.050	0.94	0.80–1.11	0.481	0.96	0.79–1.17	0.668
Subjective variables										
Pain VAS (cm)	> 1.2	5.00	4.20–5.94	< 0.001	2.63	2.12–3.26	< 0.001	–	–	–
General VAS (cm)	> 0.9	3.31	2.81–3.91	< 0.001	1.28	1.04–1.57	0.020	–	–	–
EQ5D score	0.9–1.0	Ref	–	–	Ref	–	–	–	–	–
	0.85–0.89	2.37	1.76–3.19	< 0.001	1.90	1.40–2.59	< 0.001	–	–	–
	0.75–0.84	3.90	3.14–4.84	< 0.001	2.89	2.29–3.64	< 0.001	–	–	–
	0–0.74	7.72	6.21–9.59	< 0.001	4.55	3.54–5.84	< 0.001	–	–	–
Objective variables										
TJC	≥ 1	3.13	2.64–3.71	< 0.001	–	–	–	1.75	1.43–2.13	< 0.001
SJC	≥ 1	2.82	2.38–3.34	< 0.001	–	–	–	1.41	1.15–1.73	< 0.001
Log-transformed CRP (mg/dL)	Continuous	1.29	1.22–1.36	< 0.001	–	–	–	1.10	1.04–1.17	0.002
Physician VAS (cm)	> 0.5	3.62	3.06–4.27	< 0.001	–	–	–	2.21	1.81–2.70	< 0.001
Steinbrocker stage	3 and 4	1.59	1.36–1.87	< 0.001	–	–	–	1.32	1.07–1.63	0.010
Musculoskeletal surgery	Yes	1.58	1.30–1.91	< 0.001	–	–	–	1.10	0.89–1.36	0.388
Prednisolone dose (mg)	> 5	1.99	1.39–2.84	< 0.001	–	–	–	1.25	0.86–1.82	0.245
Methotrexate use	Yes	0.75	0.63–0.97	0.018	–	–	–	0.80	0.66–0.96	0.0145
b/tsDMARDs use	Yes	0.75	0.63–0.89	< 0.001	–	–	–	0.66	0.55–0.79	< 0.001

### Factors associated with satisfaction with ADL

Compared with under 50 years old, dissatisfaction with ADL increased with aging (50–64 years) (OR 1.55 [*p* < 0.001] and OR 1.52 [*p* < 0.001]), longer disease duration (≥ 11; OR 1.25 [*p* = 0.009]), high pain VAS (OR 2.63 [*p* < 0.001]) and general VAS (OR 1.35 [*p* = 0.003]), lower EQ5D score (0.85–<0.9: OR 3.24 [*p* < 0.001]; 0.75 to < 0.85: OR 3.90 [*p* < 0.001]; < 0.75: OR 9.96 [*p* < 0.001]), presence of TJC (OR 2.02 [*p* < 0.001]) and SJC (OR 1.34 [*p* = 0.004]), higher CRP level (OR 1.10 [*p* = 0.004]), elevated VAS physician (OR 2.41 [*p* < 0.001]), Steinbrocker stages 3 and 4 (OR 1.56 [*p* < 0.001]), history of musculoskeletal surgery (OR 1.40 [*p* = 0.002]), and higher dose of prednisolone (OR 1.52 [*p* = 0.027]) (Table 4). On the other hand, the use of b/tsDMARDs increased satisfaction with ADL of RA patients (OR 0.78 [*p* = 0.008]). Additional file 1: Table S4 displays the types of musculoskeletal surgery (i.e., prosthesis, spine surgery, and others) and postoperative (< 5 years, ≥ 5 years) in each score of ADL satisfaction. The comorbidities in each score of satisfaction of ADL were shown in Additional file 1: Table S5. Sex, disease duration, VAS pain, VAS general, and EQ5D were statistically interacted in the subjective model (Table 6).

**Table 4:** Factors associated with satisfaction with activities of daily living

		Univariate			Multivariate					
					Subjective model			Objective model		
		OR	95% CI	*p*-value	OR	95% CI	*p*-value	OR	95% CI	*p*-value
Common variables										
Age (years)	< 50	Ref	–	–	Ref	–	–	Ref	–	–
	50–64	1.67	1.33–2.10	< 0.001	1.55	1.22–1.98	< 0.001	1.52	1.19–1.93	< 0.001
	65–74	1.40	1.12–1.76	0.003	1.23	0.97–1.57	0.089	1.15	0.90–1.47	0.255
	≥ 75	1.56	1.21–2.02	< 0.001	1.01	0.76–1.34	0.948	1.13	0.85–1.50	0.400
Sex	Female	1.33	1.09–1.61	0.005	1.03	0.83–1.27	0.792	1.19	0.96–1.47	0.107
Disease duration (years)	≥ 11	1.59	1.36–1.85	< 0.001	1.25	1.06–1.47	0.009	1.15	0.94–1.39	0.174
Subjective variables										
Pain VAS (cm)	> 1.2	6.15	5.18–7.30	< 0.001	2.63	2.13–3.24	< 0.001	–	–	–
General VAS (cm)	> 0.9	4.19	3.56–4.93	< 0.001	1.35	1.12–1.66	0.003	–	–	–
EQ5D socre	0.9–1.0	Ref	–	–	Ref	–	–	–	–	–
	0.85–0.89	3.96	2.94–5.33	< 0.001	3.24	2.38–4.41	< 0.001	–	–	–
	0.75–0.84	5.49	4.41–6.83	< 0.001	3.90	3.09–4.92	< 0.001	–	–	–
	0–0.74	18.11	14.37–22.81	< 0.001	9.96	7.7–12.87	< 0.001	–	–	–
Objective variables										
TJC	≥ 1	3.70	3.13–4.37	< 0.001	–	–	–	2.02	1.66–2.46	< 0.001
SJC	≥ 1	3.11	2.63–3.68	< 0.001	–	–	–	1.34	1.10–1.63	0.004
Log-transformed CRP (mg/dL)	Continuous	1.29	1.22–1.36	< 0.001	–	–	–	1.10	1.03–1.16	0.004
Physician VAS (cm)	> 0.5	4.30	3.65–5.07	< 0.001	–	–	–	2.41	1.98–2.94	< 0.001
Steinbrocker stage	3 and 4	2.21	1.89–2.59	< 0.001	–	–	–	1.56	1.26–1.92	< 0.001
Musculoskeletal surgery	Yes	2.21	1.83–2.66	< 0.001	–	–	–	1.40	1.13–1.72	0.002
Prednisolone dose (mg)	> 5	2.26	1.60–3.21	< 0.001	–	–	–	1.52	1.05–2.19	0.027
Methotrexate use	Yes	0.87	0.74–1.03	0.116	–	–	–	0.91	0.76–1.09	0.303
b/tsDMARDs use	Yes	0.98	0.83–1.15	0.773	–	–	–	0.78	0.65–0.94	0.008

### Factors associated with for satisfaction with global treatment

In comparison with patients younger than 50 years old, subjective (65–74 years: OR 0.71 [*p* = 0.007]; 75 years: OR 0.55 [*p* = 0.001]) and objective (65–74 years: OR 0.75 [*p* = 0.025]; 75 years: OR 0.64 [*p* = 0.003]) factors of satisfaction with global treatment improved with age (Table 5). Satisfaction with the overall treatment was lower in patients with high VAS pain (OR 2.16 [*p* 0.001]), low EQ5D score (0.85–0.9: OR 2.14 [*p* 0.001]; 0.75–0.85: OR 2.79 [*p* 0.001]; 0.75: OR 5.93 [*p* 0.001]), presence of TJC (OR 1.70 [*p* 0.001]) and SJC (OR 1.24 [*p* = 0.040]), and higher CRP level. The comorbidities in each score of global satisfaction were shown in Additional file 1: Table S5. Interactions in the subjective model were significantly seen for VAS pain and EQ5D, whereas those in the objective model were observed for age, Steinbrocker stage, and use of b/tsDMARDs (Table 6).

**Table 5:** Factors associated with global treatment satisfaction

		Univariate			Multivariate					
					Subjective model			Objective model		
		OR	95% CI	*p*-value	OR	95% CI	*p*-value	OR	95% CI	*p*-value
Common variables										
Age (years)	< 50	Ref	–	–	Ref	–	–	Ref	–	–
	50–64	1.15	0.91–1.45	0.236	0.99	0.77–1.26	0.917	1.03	0.81–1.32	0.795
	65–74	0.87	0.69–1.09	0.222	0.71	0.56–0.91	0.007	0.75	0.59–0.96	0.025
	≥ 75	0.87	0.66–1.13	0.284	0.55	0.41–0.73	< 0.001	0.64	0.48–0.86	0.003
Sex	Female	1.24	1.02–1.52	0.034	1.00	0.81–1.24	0.999	1.13	0.91–1.40	0.277
Disease duration (years)	≥ 11	1.28	1.09–1.49	0.002	1.06	0.89–1.25	0.508	1.13	0.93–1.38	0.225
Subjective variables										
Pain VAS (cm)	> 1.2	4.33	3.65–5.14	< 0.001	2.16	1.74–2.67	< 0.001	–	–	–
General VAS (cm)	> 0.9	3.02	2.56–3.55	< 0.001	1.18	0.96–1.45	0.115	–	–	–
EQ5D score	0.9–1.0	Ref	–	–	Ref	–	–	–	–	–
	0.85–0.89	2.51	1.85–3.39	< 0.001	2.14	1.56–2.92	< 0.001	–	–	–
	0.75–0.84	3.46	2.78–4.30	< 0.001	2.79	2.21–3.52	< 0.001	–	–	–
	0–0.74	8.60	6.90–10.71	< 0.001	5.93	4.60–7.65	< 0.001	–	–	–
Objective variables										
TJC	≥ 1	2.91	2.46–3.44	< 0.001	–	–	–	1.70	1.39–2.08	< 0.001
SJC	≥ 1	2.48	2.09–2.93	< 0.001	–	–	–	1.24	1.01–1.51	0.040
Log-transformed CRP (mg/dL)	Continuous	1.23	1.16–1.30	< 0.001	–	–	–	1.09	1.02–1.16	0.007
Physician VAS (cm)	> 0.5	3.39	2.87–4.00	< 0.001	–	–	–	2.25	1.84–2.76	< 0.001
Steinbrocker stage	3 and 4	1.56	1.33–1.83	< 0.001	–	–	–	1.15	0.93–1.42	0.198
Musculoskeletal surgery	Yes	1.51	1.25–1.83	< 0.001	–	–	–	1.09	0.87–1.35	0.446
Prednisolone dose (mg)	> 5	1.85	1.30–2.65	< 0.001	–	–	–	1.23	0.85–1.79	0.277
Methotrexate use	Yes	0.84	0.70–0.99	0.0042	–	–	–	0.84	0.69–1.00	0.055
b/tsDMARDs use	Yes	1.07	0.91–1.27	0.423	–	–	–	0.93	0.78–1.13	0.93

**Table 6:** Results of the instrumental variable analysis for each aspect of satisfaction

Satisfaction	Model	Interacted factors	*p*-value for interaction
Cost of treatment	Subjective model	Disease duration and EQ5D	< 0.001
		Pain VAS and EQ5D	0.004
	Objective model	Age and b/tsDMARDs use	0.011
		Sex and log-transformed CRP	< 0.001
		Disease duration and history of musculoskeletal surgery	0.011
		Log-transformed CRP and physician VAS	0.012
		History of musculoskeletal surgery and methotrexate use	0.026
		History of musculoskeletal surgery and b/tsDMARDs use	< 0.001
Effect of treatment	Subjective model	Disease duration and EQ5D	0.007
		General VAS and EQ5D	0.013
	Objective model	TJC and history of musculoskeletal surgery	0.020
		Age and methotrexate use	0.031
		SJC and methotrexate use	0.002
		Physician VAS and methotrexate use	< 0.001
ADL	Subjective model	Disease duration and sex	0.017
		Pain VAS and EQ5D	0.024
		General VAS and EQ5D	0.030
	Objective model	History of musculoskeletal surgery and Steinbrocker stage	0.004
		Methotrexate use and b/tsDMARDs use	0.022
Global treatment	Subjective model	General VAS and EQ5D	< 0.001
	Objective model	Age and Steinbrocker stage	0.022
		Age and b/tsDMARDs use	0.005
		TJC and physician VAS	0.004

## Discussion

Because there are currently no large cohort studies investigating patient satisfaction focusing on the cost of treatment, we used the data of 2235 RA patients to separately evaluate patient satisfaction regarding different aspects (i.e., cost of treatment, treatment effect, ADL, and global treatment). On multivariate analysis, we discovered several patient-related factors influencing each aspect of satisfaction. Because recent T2T treatments have resulted in low disease activity and remission, the majority of FRANK Registry participants had low disease activity and was remission. Good disease control has also revealed that they were “very satisfied” or “satisfied” with their current treatment in terms of cost and effectiveness, ADL, and global treatment upon initial registration, yet there were still “somewhat satisfied,” “somewhat unsatisfied,” “unsatisfied,” and “very unsatisfied” patients in each satisfaction (Fig. 2). As even some patients achieving low disease activity or remission have remained symptoms, so-called an unmet need, especially concerning improving pain, fatigue, and function [31], the factors causing the dissatisfaction have been required to elucidate in treatment for RA. Although few studies have examined RA patient satisfaction in large cohorts, a German biological register study of 10,646 RA patients using b/tsDMARDs discovered that tapering glucocorticoids was positively associated with patient satisfaction [10]. Furthermore, because treatment costs were negatively related to satisfaction [14–16] and because the health care costs of RA patients are higher than those of controls matched for age, sex, and medical history [32], it is important to understand their satisfaction regarding the cost of long periods of RA treatment. In a Chinese registry of RA, patients with a low proportion of treatment cost to income were more satisfied than those with a high proportion [17]. As a result, we looked into patient satisfaction with the cost of treatment separately. Aging, a high EQ5D score, the absence of SJC, an elevated serum CRP level, a history of musculoskeletal surgery, and nonuse of b/tsDMARDs were all associated with increased cost satisfaction (Table 2). On the other hand, factors like aging, higher disease activity (each VAS, presence of TJC or SJC, elevated serum CRP level), Steinbrocker stages 3 and 4, a history of musculoskeletal surgery, and higher prednisolone dose were related to lower satisfaction with treatment, ADL, and global treatment.

We found that cost of satisfaction increased with aging in both subjective and objective models of the multivariate analysis. In the Japanese health care system, the government typically covers 70% of treatment costs, with patients responsible for the remaining 30%. However, co-payment has been adjusted to 10–30% depending on the family’s income and age (< 65 years, 30%; 65–74 years, 20%; ≥ 75 years, 10% co-payment). As a result, because older patients pay a lower percentage of co-payment than younger patients, cost satisfaction may markedly increase with age. The statistical significance of the interaction was seen in age and b/tsDMARDs using in the satisfaction of cost of treatment (Table 6), which suggests that satisfaction cost of treatment affected by b/tsDMARDs was modified by aging. For this reason, the satisfaction of cost in b/tsDMARDs might be different with aging. In addition, patients with a history of musculoskeletal surgery were more satisfied with the cost of treatment. The rate of patients with the operation of the prosthesis was higher in “very satisfied” (21%) and “satisfied” (13%) of satisfaction for cost, and there was no difference in the patients who undergone spine surgery and other surgeries (Additional file 1: Table S3). However, the rate of patients undertaking prostheses was increased due to dissatisfaction with ADL (Additional file 1: Table S4). Taken together, patients undertaking musculoskeletal surgery might feel disability, and patients with disabilities are given medical coverage by the government, suggesting that patients with a history of musculoskeletal surgery might have increased satisfaction with cost due to the presence of some disabilities. Patients, who had musculoskeletal surgery more than 5 years ago, have accounted for more than half of this cohort, and there was no difference in the distribution of cost satisfaction and ADL (Table 1 and Additional file 1: Tables S3 and S4). The history of musculoskeletal surgery has also shown the interaction with disease duration or b/tsDMARDs use (Table 6), suggesting that the musculoskeletal surgery on the satisfaction of cost might be affected with disease duration or use of b/tsDMARDs. QOL of RA patients was evaluated by EQ5D score [33]. In agreement with previous evidence [7, 13, 34, 35], the EQ5D score in our cohort also affected all aspects of satisfaction (treatment cost, efficacy, ADL, and global treatment), indicating that the improvement of EQ5D should be critical for better RA treatment and patient satisfaction. Because EQ5D showed interacted with disease duration, VAS pain, and VAS general in terms of treatment cost, treatment effect, ADL, and global treatment, the satisfaction associated with EQ5D can be separately assessed by each factor. Treatment with b/tsDMARDs improves treatment outcomes and QOL in RA patients [36–38], and the T2T strategy with b/tsDMARDs is recommended. Better QOL and the use of b/tsDMARDs have been shown to be positive predictors of overall treatment satisfaction. Indeed, the use of b/tsDMARDs increased satisfaction regarding treatment effect and ADL in our cohort but decreased cost satisfaction significantly. In satisfaction of cost of treatment in our study, the rate of methotrexate use was slightly lower in patients with “unsatisfied” and “very unsatisfied;” on the other hand, the rate of b/tsDMARDs use was elevated depending on unsatisfaction (Additional file 1: Table S2). Because the high cost of b/tsDMARDs is a burden on health care budgets, the use of biosimilars has been widely approved with reported benefits, such as cost-effectiveness [39–44]. However, our cohort consisted only a few biosimilars. As a result, the increased use of biosimilars may have an impact on cost satisfaction in our cohort. Because the use of b/tsDMARDs interacted with age and history of musculoskeletal surgery in terms of satisfaction with cost and global treatment (Table 6). These factors may have an impact on these aspects of satisfaction. Methotrexate, the main treatment for RA, affected satisfaction for the effect of treatment but had less effect for satisfaction for cost, ADL, and global treatment. In a previous study compared with bDMARDs and methotrexate for treatment with juvenile idiopathic arthritis, bDMARDs increased satisfaction more than MTX [45]. Taken together, our finding also suggested that the use of b/tsDMARDs improved patient satisfaction more than methotrexate except for satisfaction for cost.

According to several studies, disease activity influences treatment effectiveness and patient satisfaction, whereas treatment cost is an independent factor of treatment satisfaction [10, 12, 13, 46]. High disease activity, in particular, reduces treatment satisfaction. In our cohort, disease activity, including SJC, TJC, each VAS, and serum CRP level, correlated with increased dissatisfaction with treatment effect, ADL, and overall treatment. Only elevated serum CRP levels, on the other hand, reduced dissatisfaction with treatment costs. The statistical significance of the interaction was seen in log-transformed CRP and sex or physician VAS in the satisfaction of cost of treatment (Table 6), indicating that satisfaction of cost of treatment affected by CRP level was influenced by sex or physician VAS. Serum CRP level and medical therapy (i.e., use and dose of prednisolone, and dose of methotrexate) in each score of satisfaction of cost of treatment have shown no difference (Additional file 1: Table S2), suggesting that the reason for the higher satisfaction of cost related with elevated CRP levels was not due to lower intake and cost of medication. Although this study could not determine the reason, the median (0.11 mg/dL) and average ± standard deviation (0.40 ± 1.0 mg/dL) of serum CRP level in our cohort were low, indicating that slightly increased serum CRP level might provide good cost performance for RA patients.

Schäfer et al. [10] found that tapering prednisolone was associated with higher levels of satisfaction in RA patients taking b/tsDMARDs. Prednisolone treatment, particularly low doses, is safe and effective [47], but continuous use of higher doses should be avoided due to the risk of infection, osteoporosis, diabetes mellitus, thrombotic stroke, cardiovascular disease, and death [48–51]. In a recent study [47], however, only nearly half of 508 patients with early RA were able to discontinue prednisolone; inability to taper prednisolone has been reported as one of the characteristics of difficult-to-treat RA [52]. In this study, less than half of the patients with RA (40%) were treated with prednisolone to control disease activity, suggesting that these patients might have difficult-to-treat RA. This may imply a decreased capacity to perform ADL, indicating that the use of higher doses of prednisolone led to decreased ADL satisfaction.

Surprisingly, differences in satisfaction were revealed as people aged. We discovered that as people aged, their treatment costs and overall satisfaction increased, but their treatment effect and ADL satisfaction decreased. Previous studies have shown lower EQ5D scores with aging due to poorer physical function and capacity to perform ADL [53, 54], leading to decreased life satisfaction. In our study, satisfaction with treatment effect and ADL was lower in the age group of 50–64 years old in particular. The lower cost of treatment and disease activity in our cohort may have increased global treatment satisfaction with aging. Previous research has shown that a longer disease duration of RA was associated with impaired ADL [55]. Similarly, in our study, longer disease duration and higher Steinbrocker stage were associated with lower ADL satisfaction.

There are several limitations to this study. First, patient satisfaction in this study was evaluated using a Likert scale instead of a validated scoring system, such as the treatment satisfaction questionnaire for medication (TSQM) [56]. As the FRANK longitudinal registry has been collected in the outpatient department every year, it makes it difficult to fill numerous questionnaires each time. These separate simple questionnaires on the cost of treatment, the effect of treatment, ADL, and global treatment established the differentiation of each satisfaction. Secondly, because this was a cross-sectional study using data from the initial registrants of the FRANK Registry, the changes of variables in satisfaction were unclear. Further study is needed to elucidate these changes by accumulating the registered longitudinal follow-up data in this large cohort of Japanese RA patients. Moreover, our study could show the meaningful outcome affecting various satisfactions by using a large cohort. Third, our cohort consisted primarily of patients in remission or with low disease activity. Because of the widespread use of csDMARDs and bDMARDs, the T2T strategy has resulted in a greater number of patients achieving lower disease activity. However, there are still patients who are dissatisfied with the effect of treatment, cost of treatment, ADL, and global treatment, and thus, the analysis of satisfaction in RA patients is important and meaningful to further improve their treatment. Actually, the rates of the use of methotrexate and b/tsDMARDs were 72% and 31% each. Finally, the duration of the last treatment might be possible to affect each satisfaction; however, this study did not include the analysis of the time of last treatment in the medication. In this initial registration, we have only obtained the content of past b/tsDMARDs, but not the duration of their last medication because the majority of the registered patients had a long history of treatment. Since this observational cohort study has accumulated the history of medication every year after initial registration, we could analyze the relationship with each satisfaction and the duration of medication hereafter.

## Conclusion

We have conducted a large cohort observational study using patients in the FRANK Registry in the south of Japan. We assessed patient satisfaction in terms of cost, treatment effect, ADL, and global treatment and identified the factors influencing each of these aspects. Aging was linked to higher costs and overall treatment satisfaction, and global treatment satisfaction, but also with decreased treatment and ADL satisfaction. Female sex and having a history of musculoskeletal surgery improved cost satisfaction, whereas a lower EQ5D score and higher disease activity were associated with lower overall satisfaction. Using b/tsDMARDs reduced cost satisfaction while increasing treatment effect and ADL satisfaction.

## Supplementary Information

**Additional file 1: Table S1.** The correlation coefficients for each satisfaction. **Table S2.** Serum CRP levels and medication of each score of satisfaction of cost of treatment at initial registration. **Table S3.** The kinds of musculoskeletal surgery (i.e., prosthesis, spine surgery, and others) and the postoperative duration (5 years>, 5 years≤) in each score of satisfaction of cost of treatment at initial registration. Others included arthrodesis, arthroplasty, synovectomy, surgery for tendon, or surgery associated with fracture. **Table S4.** The kinds of musculoskeletal surgery (i.e., prosthesis, spine surgery, and others) and the postoperative duration (5 years>, 5 years≤) in each score of satisfaction of ADL at initial registration. Others included arthrodesis, arthroplasty, synovectomy, surgery for tendon, or surgery associated with fracture. **Table S5.** The comorbidities (i.e., cardiovascular disorder, pulmonary disorder, and osteoporosis) in each score of satisfaction (i.e., cost of treatment, effect of treatment, ADL, and global treatment) at initial registration.

## Data Availability

The datasets generated and/or analyzed during the current study are not publicly available.

## Abbreviations

DMARD: Disease-modifying anti-rheumatic drug
RA: Rheumatoid arthritis
ADL: Activity of daily living
EQ5D: EuroQol 5-Dimensional Questionnaire
QOL: Quality of life
T2T: Treat-to-target
FRANK: Fukuoka Rheumatoid Arthritis Network
VAS: Visual analog scales
TJC: Tender 28-joint count
SJC: Swollen 28-joint count
CRP: C-reactive protein
ESR: Erythrocyte sedimentation ratio
mHAQ: Modified Health Assessment Questionnaire
OR: Odds ratio
