# Supplementary material for: Factors affecting patient satisfaction related to cost and treatment effectiveness in rheumatoid arthritis: results from the multicenter observational cohort study, FRANK Registry
Source: Arthritis Res Ther. 2022 Feb 22;24:53. doi: 10.1186/s13075-022-02746-5 (PMC8862466; doi:10.1186/s13075-022-02746-5)
Supplement: Supplementary file 1 — Additional file 1: Table S1. The correlation coefficients for each satisfaction. Table S2. Serum CRP levels and medication of each score of satisfaction of cost of treatment at initial registration. Table S3. The kinds of musculoskeletal surgery (i.e., prosthesis, spine surgery, and others) and the postoperative duration (5 years>, 5 years≤) in each score of satisfaction of cost of treatment at initial registration. Others included arthrodesis, arthroplasty, synovectomy, surgery for tendon, or surgery associated with fracture. Table S4. The kinds of musculoskeletal surgery (i.e., prosthesis, spine surgery, and others) and the postoperative duration (5 years>, 5 years≤) in each score of satisfaction of ADL at initial registration. Others included arthrodesis, arthroplasty, synovectomy, surgery for tendon, or surgery associated with fracture. Table S5. The comorbidities (i.e., cardiovascular disorder, pulmonary disorder, and osteoporosis) in each score of satisfaction (i.e., cost of treatment, effect of treatment, ADL, and global treatment) at initial registration. [file 13075_2022_2746_MOESM1_ESM.docx]

Supplemental Table 1: The correlation coefficients for each satisfaction

|  | Satisfaction for cost of treatment | Satisfaction for effect of treatment | Satisfaction for ADL | Satisfaction for global treatment |
| --- | --- | --- | --- | --- |
| Satisfaction for cost of treatment | r=1.00 | r=0.32 | r=0.25 | r=0.43 |
| Satisfaction for effect of treatment | r=0.32 | r=1.00 | r=0.69 | r=0.76 |
| Satisfaction for ADL | r=0.25 | r=0.69 | r=1.00 | r=0.74 |
| Satisfaction for global treatment | r=0.43 | r=0.43 | r=0.74 | r=1.00 |

Spearman Correlation Coefficients

Supplemental Table 2: Serum CRP levels and medication of each score of satisfaction of cost of treatment at initial registration

|  | score of satisfaction of cost of treatment | | | | | |
| --- | --- | --- | --- | --- | --- | --- |
|  | Very satisfied | Satisfied | Somewhat satisfied | Somewhat unsatisfied | Unsatisfied | Very unsatisfied |
| Serum CRP level (mg/dL) (median, range) | 0.2 (0-16.8) | 0.1 (0-16.5) | 0.1 (0-4.3) | 0.1 (0-21.9) | 0.1 (0-11.0) | 0.1 (0-4.3) |
| Prednisolone use (yes) (%) | 125 (47%) | 302 (37%) | 246 (42%) | 159 (43%) | 45 (35%) | 19 (32%) |
| Prednisolone dose (mg) (median, range) | 3 (1-10) | 4 (1-13) | 3 (0-12) | 4 (1-15) | 5 (0-18) | 4 (1-8) |
| Methotrexate use (yes) (%) | 197 (74%) | 584 (71%) | 439 (75%) | 274 (74%) | 83 (64%) | 37 (62%) |
| Methotrexate dose (mg) (median, range) | 8 (2-20) | 8 (2-16) | 8 (2-16) | 8 (2-16) | 8 (2-16) | 8 (2-14) |
| b/tsDMARDs use (yes) (%) | 86 (32%) | 172 (21%) | 142 (24%) | 164 (44%) | 84 (65%) | 49 (82%) |

Supplemental Table 3: The kinds of musculoskeletal surgery (i.e., prosthesis, spine surgery, and others) and the postoperative duration (5 years>, 5 years≤) in each score of satisfaction of cost of treatment at initial registration

|  | score of satisfaction of cost of treatment | | | | | |
| --- | --- | --- | --- | --- | --- | --- |
|  | Very satisfied  (N=268) | Satisfied  (N=819) | Somewhat satisfied  (N=589) | Somewhat unsatisfied  (N=370) | Unsatisfied  (N=129) | Very unsatisfied  (N=60) |
| Prosthesis | 55 (21%) | 105 (13%) | 55 (9%) | 18 (5%) | 10 (8%) | 3 (5%) |
| 5 years> (%) | 8 (15%) | 35 (33%) | 19 (35%) | 9 (50%) | 4 (40%) | 2 (67%) |
| 5 years≤ (%) | 47 (85%) | 70 (67%) | 36 (65%) | 9 (50%) | 6 (60%) | 1 (33%) |
| Spine surgery | 12 (4%) | 22 (3%) | 7 (1%) | 4 (1%) | 3 (2%) | 0 (0%) |
| 5 years> (%) | 3 (25%) | 9 (41%) | 1 (14%) | 1 (25%) | 1 (33%) | 0 (0%) |
| 5 years≤ (%) | 9 (75%) | 13 (59%) | 6 (86%) | 3 (75%) | 2 (67%) | 0 (0%) |
| Others | 35 (13%) | 73 (9%) | 33 (6%) | 17 (5%) | 8 (6%) | 6 (10%) |
| 5 years> (%) | 8 (23%) | 26 (36%) | 11 (34%) | 8 (47%) | 5 (63%) | 3 (50%) |
| 5 years≤ (%) | 27 (77%) | 47 (64%) | 21 (66%) | 9 (53%) | 3 (38%) | 3 (50%) |

Others included arthrodesis, arthroplasty, synovectomy, surgery for tendon, or surgery associated with fracture.

Supplemental Table 4: The kinds of musculoskeletal surgery (i.e., prosthesis, spine surgery, and others) and the postoperative duration (5 years>, 5 years≤) in each score of satisfaction of ADL at initial registration

|  | score of satisfaction of ADL of treatment | | | | | |
| --- | --- | --- | --- | --- | --- | --- |
|  | Very satisfied  (N=400) | Satisfied  (N=887) | Somewhat satisfied  (N=600) | Somewhat unsatisfied  (N=244) | Unsatisfied  (N=90) | Very unsatisfied  (N=14) |
| Prosthesis | 11 (3%) | 86 (10%) | 86 (14%) | 36 (15%) | 24 (27%) | 3 (21%) |
| 5 years> | 4 (36%) | 26 (30%) | 30 (35%) | 12 (33%) | 3 (13%) | 2 (67%) |
| 5 years≤ | 7 (64%) | 60 (70%) | 56 (65%) | 24 (67%) | 21 (88%) | 1 (33%) |
| Spine surgery | 9 (2%) | 13 (1%) | 15 (3%) | 7 (3%) | 3 (3%) | 1 (7%) |
| 5 years> | 2 (22%) | 4 (31%) | 4 (27%) | 3 (43%) | 1 (33%) | 1 (100%) |
| 5 years≤ | 7 (78%) | 9 (69%) | 11 (73%) | 4 (57%) | 2 (67%) | 0 (0%) |
| Others | 10 (3%) | 53 (6%) | 61 (10%) | 29 (12%) | 17 (19%) | 1 (7%) |
| 5 years> | 3 (30%) | 22 (42%) | 24 (39%) | 7 (24%) | 5 (29%) | 0 (0%) |
| 5 years≤ | 7 (70%) | 31 (58%) | 37 (61%) | 22 (76%) | 12 (71%) | 1 (100%) |

Others included arthrodesis, arthroplasty, synovectomy, surgery for tendon, or surgery associated with fracture.

Supplemental Table 5: The comorbidities (i.e., cardiovascular disorder, pulmonary disorder, and osteoporosis) in each score of satisfaction (i.e., cost of treatment, effect of treatment, ADL, and global treatment) at initial registration

|  |  | Comorbidities | | |
| --- | --- | --- | --- | --- |
|  |  | Cardiovascular disorder  (N=106) | Pulmonary disorder  (N=193) | Osteoporosis  (N=285) |
| Satisfaction of cost of treatment | Very satisfied (%) | 26 (5%) | 36 (7%) | 55 (10%) |
|  | Satisfied (%) | 47 (4%) | 88 (8%) | 147 (14%) |
|  | Somewhat satisfied (%) | 25 (5%) | 50 (10%) | 59 (12%) |
|  | Somewhat unsatisfied (%) | 6 (5%) | 14 (11%) | 20 (16%) |
|  | Unsatisfied (%) | 2 (7%) | 5 (17%) | 4 (14%) |
|  | Very unsatisfied (%) | 0 (0%) | 0 (0%) | 0 (0%) |
| Satisfaction of effect of treatment | Very satisfied (%) | 16 (6%) | 20 (7%) | 35 (13%) |
|  | Satisfied (%) | 49 (6%) | 75 (9%) | 134 (16%) |
|  | Somewhat satisfied (%) | 21 (4%) | 51 (9%) | 66 (11%) |
|  | Somewhat unsatisfied (%) | 14 (4%) | 35 (9%) | 33 (9%) |
|  | Unsatisfied (%) | 5 (4%) | 10 (8%) | 7 (5%) |
|  | Very unsatisfied (%) | 1 (2%) | 2 (3%) | 10 (17%) |
| Satisfaction of ADL | Very satisfied (%) | 18 (5%) | 22 (6%) | 36 (9%) |
|  | Satisfied (%) | 40 (5%) | 76 (9%) | 121 (14%) |
|  | Somewhat satisfied (%) | 34 (6%) | 56 (9%) | 84 (14%) |
|  | Somewhat unsatisfied (%) | 10 (4%) | 25 (10%) | 33 (14%) |
|  | Unsatisfied (%) | 4 (4%) | 14 (16%) | 10 (11%) |
|  | Very unsatisfied (%) | 0 (0%) | 0 (0%) | 1 (7%) |
| Satisfaction of global treatment | Very satisfied (%) | 23 (6%) | 25 (7%) | 41 (11%) |
|  | Satisfied (%) | 50 (5%) | 89 (8%) | 156 (14%) |
|  | Somewhat satisfied (%) | 27 (5%) | 58 (10%) | 64 (11%) |
|  | Somewhat unsatisfied (%) | 4 (2%) | 18 (11%) | 18 (11%) |
|  | Unsatisfied (%) | 2 (6%) | 2 (6%) | 6 (18%) |
|  | Very unsatisfied (%) | 0 (0%) | 1 (33%) | 0 (0%) |
